# Overseas Treatment of Latent Tuberculosis Infection in US–Bound Immigrants

**DOI:** 10.3201/eid2803.212131

**Published:** 2022-03

**Authors:** Amera Khan, Christina R. Phares, Hoang Lan Phuong, Dang Thi Kieu Trinh, Ha Phan, Cindy Merrifield, Phan Thi Hong Le, Quach Thi Kim Lien, Sooc Ngoc Lan, Phan Thi Kim Thoa, Le Tran Minh Thu, Tiffany Tran, Cuc Tran, Lucy Platt, Susan A. Maloney, Nguyen Viet Nhung, Payam Nahid, John E. Oeltmann

**Affiliations:** Stop TB Partnership, Geneva, Switzerland (A. Khan); London School of Hygiene and Tropical Medicine, London, UK (A. Khan, L. Platt);; Centers for Disease Control and Prevention, Atlanta, Georgia, USA (C.R. Phares, C. Tran, S.A. Maloney, J.E. Oeltmann);; Cho Ray Hospital Visa Medical Clinic, Ho Chi Minh City, Vietnam (H.L. Phuong, D.T.K. Trinh, P.T.H. Le, Q.T.K. Lien, S.N. Lan, P.T.K. Thoa, L.T.M. Thu);; Vietnam National TB Program/University of California–San Francisco Research Collaboration, Hanoi, Vietnam (H. Phan, C. Merrifield, T. Tran, N.V. Nhung, P. Nahid);; University of California–San Francisco, San Francisco, California, USA (H. Phan, C. Merrifield, P. Nahid);; Vietnam National TB Program, Hanoi (N.V. Nhung)

**Keywords:** tuberculosis and other mycobacteria, respiratory infections, bacterial infections, United States, bacteria

## Abstract

Seventy percent of tuberculosis (TB) cases in the United States occur among non–US-born persons; cases usually result from reactivation of latent TB infection (LTBI) likely acquired before the person’s US arrival. We conducted a prospective study among US immigrant visa applicants undergoing the required overseas medical examination in Vietnam. Consenting applicants >15 years of age were offered an interferon-γ release assay (IGRA); those 12–14 years of age received an IGRA as part of the required examination. Eligible participants were offered LTBI treatment with 12 doses of weekly isoniazid and rifapentine. Of 5,311 immigrant visa applicants recruited, 2,438 (46%) consented to participate; 2,276 had an IGRA processed, and 484 (21%) tested positive. Among 452 participants eligible for treatment, 304 (67%) initiated treatment, and 268 (88%) completed treatment. We demonstrated that using the overseas medical examination to provide voluntary LTBI testing and treatment should be considered to advance US TB elimination efforts.

In 1989, the US Advisory Council on the Elimination of Tuberculosis declared a goal to eliminate tuberculosis (TB) in the United States by 2010 (*1*). TB elimination is defined as <1 case/1 million population; in 2018, the United States reported 28 TB cases/1 million population (*1*). Although US TB incidence has been declining for the past 20 years, with an all-time low of ≈9,000 reported cases in 2018, TB elimination is still far from reality (*2*).

US TB epidemiology can be summarized as a dwindling overall incidence with an increasing proportion of cases diagnosed among non–US-born persons. In 2018, 70.2% of TB cases were diagnosed among non–US-born persons (*2*). Molecular studies suggest most TB cases occurring among non–US-born persons are caused by reactivation of latent TB infection (LTBI), likely acquired before the person’s US arrival because of higher risk for TB exposure overseas (*3*,*4*). LTBI treatment has been demonstrated to substantially reduce the risk for progression to TB disease (*5*). Modeling studies suggest progression toward TB elimination requires strengthening efforts for diagnosing and treating LTBI among non–US-born persons (*6*,*7*). However, postarrival stateside strategies to address LTBI have had suboptimal results (*8*). A recent analysis of data on newly arriving immigrants and refugees at risk for TB found that 35.5% did not complete a US postarrival evaluation for TB and LTBI. Among those who did and were recommended for LTBI treatment, 69.0% initiated treatment and 40.0% completed treatment (*8*).

Immigrant visa applicants abroad are required to undergo a medical examination before US arrival, conducted by panel physicians who are under agreement with the US Department of State. The purpose of the overseas medical examination is to screen for communicable diseases of public health importance as required by the US Immigration and Nationality Act (8 US Code 1182 and 1222) and the Public Health Service Act (US Code 252). Because TB is transmissible, screening and treatment for TB disease are essential components of the examination and are performed in accordance with the Centers for Disease Control and Prevention (CDC) Technical Instructions for Tuberculosis Screening and Treatment Using Cultures and Directly Observed Therapy 2019 (*9*). Improvements in TB screening and treatment in the overseas medical examination have been associated with a temporal decline in TB cases among non–US-born persons in the United States since 2007 (*10*).

One potential strategy to improve the uptake and completion of LTBI treatment among non–US-born persons is to expand the overseas medical examination to include the use of IGRAs to identify persons with TB infection, and to offer a voluntary 3-month regimen of isoniazid and rifapentine given once weekly (3HP) treatment to applicants who had LTBI diagnosed before immigration (*11*). To date, empirical evidence on the feasibility, acceptability, and effectiveness of predeparture testing and treatment approach is scarce. Therefore, we conducted a prospective study to assess voluntary uptake of LTBI testing and 3HP treatment initiation and completion among US-bound immigrant visa applicants in Vietnam while following them through the LTBI cascade of care.

## Methods

Vietnam is in the top 5 countries of birth for non–US-born persons with TB in the United States (*2*). According to the World Health Organization, Vietnam has a high TB burden, with an incidence of 182 cases/100,000 population (95% CI 116–263 cases/100,000 population) (*12*). Approximately one third of the adult population in Vietnam has LTBI (*13*). The Cho Ray Hospital Visa Medical Department (CRH VMD) in Ho Chi Minh City, the main panel physician site, screens ≈1,500 US-bound immigrant visa applicants per month and was selected as the study site.

During September 2018–October 2019, we conducted a prospective study, the Preventing Tuberculosis Overseas Pilot Study (PTOPS), at CRH VMD. Our aim was to assess voluntary uptake of LTBI testing and treatment initiation and completion by US-bound immigrant visa applicants.

### Study Eligibility Criteria

Study eligibility included US-bound immigrant visa applicants attending their required medical examination who were >12 years of age, not pregnant or breastfeeding, and living in the area of Ho Chi Minh City Province. If during the medical examination or study participants were found to have any of the following conditions, they were excluded from participation: signs or symptoms of TB disease, HIV infection, close-contact with someone with isoniazid- or rifampin-resistant TB; previous treatment for TB disease or LTBI; substance-related disorders or mental disorders; sensitivity to isoniazid or rifamycins; or hepatitis B or C. Participants were also excluded from the study if they had a baseline serum alanine aminotransferase (serum glutamic pyruvic transaminase) >5× the upper limit of normal. Those with known liver disease were excluded if they had a baseline alanine aminotransferase >3× the upper limit of normal or total bilirubin >2× the upper limit of normal.

### Study Process

**Figure 1:**
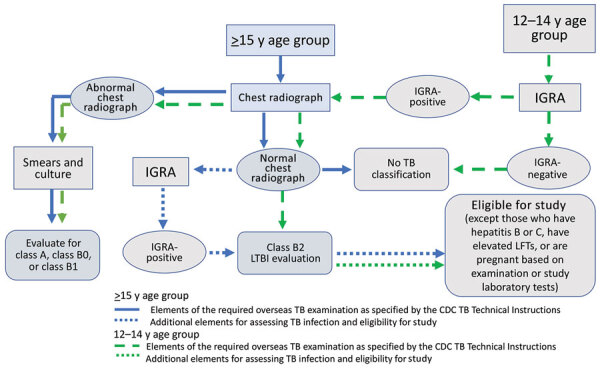
Overseas TB medical examination pathway and TB classifications for US immigrant visa applicants and modifications for the Preventing Tuberculosis Overseas Pilot Study, Vietnam, 2018–2019. TB classifications for overseas medical examination outcomes: No TB classification, no TB disease or infection; class A, TB disease (treatment completion required); class B0, completed treatment for TB disease by directly observed therapy supervised by panel physician; class B1, clinical signs, symptoms, or chest radiograph suggestive of TB or known HIV infection but negative sputum smears and culture; class B2, LTBI evaluation. CDC, Centers for Disease Control and Prevention; IGRA, interferon-γ release assay; LFTs, liver function tests; LTBI, latent tuberculosis infection; TB, tuberculosis.

Ethical approvals were obtained by CDC, London School of Hygiene and Tropical Medicine, University of California–San Francisco, and the Vietnam National Lung Hospital. During recruitment, eligible immigrant visa applicants were provided study information in Vietnamese and an opportunity to ask questions (Figure 1). Applicants were informed that participation was voluntary and accepting or declining to participate would not impact their visa application. They were also informed that LTBI testing and treatment are available in the United States after arrival, if they preferred. For insight into losses in the LTBI care cascade, those who declined to participate were asked if they would be willing to provide their reasons for nonparticipation. Immigrant visa applicants who consented to participate were enrolled (Table 1). Participants >15 years of age were administered an IGRA, the QuantiFERON Gold in Tube Test (QIAGEN, https://www.qiagen.com) to test for TB infection free of charge as part of the study. Participants 12–14 years of age were already required to receive an IGRA as part of the medical examination. Additional laboratory tests for participants included liver function tests, hepatitis B and C serologic test, and pregnancy tests, if indicated. All laboratory tests were processed unless participants withdrew from the study or were determined to be ineligible because of an abnormal chest radiograph or any other signs or symptoms of TB disease discovered. 

**Table 1:** Characteristics of study participants in the Preventing Tuberculosis Overseas Pilot Study of US immigrant visa applicants, Vietnam, 2018–2019*

Characteristic	No. (%) participants						
	Recruited	Enrolled	IGRA processed	IGRA-positive	3HP-eligible	Initiated 3HP	Completed 3HP
Total	5,311	2,438	2,276	484	452	304	268
Sex							
F	2,888 (54)	1,350 (55)	1,304 (57)	272 (56)	259 (57)	170 (56)	152 (57)
M	2,423 (46)	1,088 (45)	972 (43)	212 (44)	193 (43)	134 (44)	116 (43)
Age group, y							
12–14	298 (6)	143 (6)	142 (6)†	9 (2)	9 (2)	4 (1)	4 (1)
15–17	431 (8)	226 (9)	223 (10)	19 (4)	18 (4)	14 (5)	13 (5)
18–35	1,527 (29)	773 (32)	749 (33)	114 (24)	109 (24)	69 (23)	62 (23)
36–65	2,909 (55)	1,254 (51)	1,128 (50)	333 (69)	307 (68)	211 (69)	184 (69)
≥66	146 (3)	42 (2)	34 (1)	9 (2)	9 (2)	6 (2)	5 (2)

*IGRA, interferon-γ release assay; 3HP, 3-mo regimen of isoniazid and rifapentine. †IGRA required as part of medical examination for participants 12–14 y of age; 1 participant’s IGRA was not processed for the study because of recent measles, mumps, and rubella vaccination.

For IGRA-negative participants and for IGRA-positive participants who were also hepatitis B- or C-positive, pregnant, or otherwise ineligible, no further participation in the study was requested. However, as part of the immigration process, these IGRA-positive participants were categorized with a class B2 TB, LTBI evaluation classification, which alerts US health departments through CDC’s Electronic Disease Notification (EDN) system of the arrival of persons with LTBI (*14*). The classification comes with the recommendation for immigrants to complete a postarrival follow-up evaluation at a US health department where LTBI treatment can be provided if indicated.

The remaining IGRA-positive participants were offered 3HP (12 weekly doses) by directly observed therapy (DOT) at CRH VMD free of charge as part of the study. For those emigrating to the United States before treatment completion, an option was provided for completing >8 doses of DOT at CRH VMD and taking the remaining <4 doses by self-administration therapy (SAT) in the United States. Thus, a minimum of 8 weeks’ stay in Vietnam before immigration was required for participation in the treatment portion of the study. IGRA-positive participants who declined treatment were asked to provide their reasons for declining and were educated about the signs and symptoms of TB. They received a B2 classification with the recommendation to complete a postarrival follow-up in the United States. Participants who accepted treatment were given 3HP weekly by DOT for at least the first 8 doses at CRH VMD. At these DOT visits, participants were assessed for treatment side effects. Those who took the last <4 doses by SAT in the United States received a weekly follow-up call by a US-based, Vietnamese-speaking study coordinator to document whether treatment was taken and to assess for any adverse events. We defined treatment completion as taking >11 of the 12 doses of 3HP within 16 weeks (*15*).

## Results

### Study Flow and LTBI Cascade of Care

**Figure 2:**
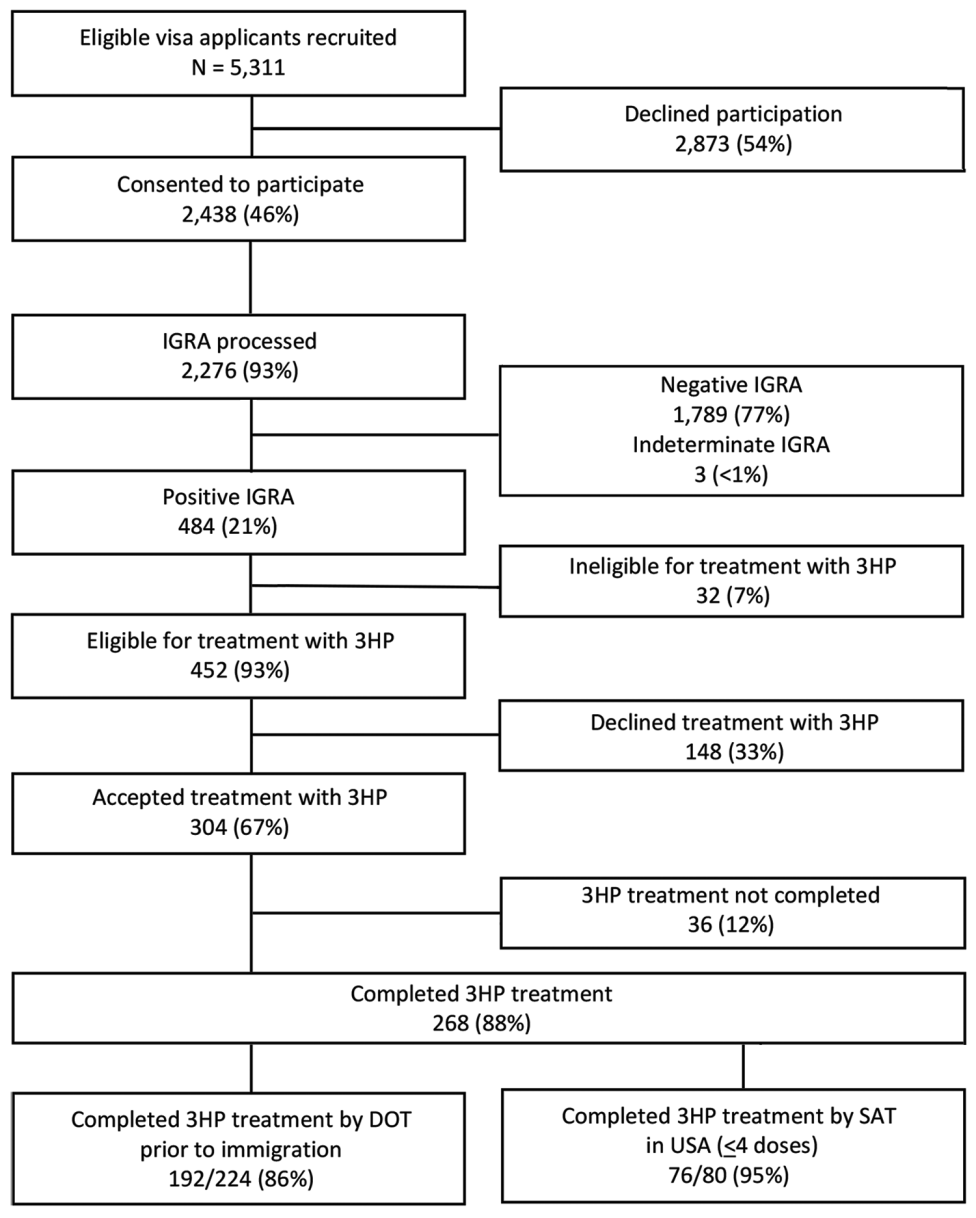
Flowchart of US immigrant visa applicants who consented to participate, initiated treatment, and completed treatment along the latent TB infection cascade of care in the Preventing Tuberculosis Overseas Pilot Study, Vietnam, 2018–2019. Participants who completed ≥8 doses of 3HP by DOT in Vietnam were given the option of taking the remaining ≤4 doses by SAT after arrival in the United States. DOT, directly observed therapy; IGRA, interferon-γ release assay; LTBI, latent tuberculosis infection; SAT, self-administered therapy; TB, tuberculosis; 3HP, 3-month regimen of isoniazid and rifapentine.

Of 5,311 eligible US-bound immigrant visa applicants, 2,438 (46%) consented to participate in the study and receive an IGRA to test for LTBI (including 143 applicants 12–14 years of age for whom an IGRA was a required component of their immigrant medical examination) (Figure 2). Among those who consented, 2,276 (93%) received an IGRA and additional study laboratory tests; the remaining 7% who had consented either withdrew from the study or were found to be ineligible during the medical examination before the processing of the IGRA. Among participants who received an IGRA, 484 (21%) were positive and 452 were eligible for 3HP. Of those who were eligible, 304 (67%) initiated treatment and 268 (88%) successfully completed treatment; 192 (72%) persons completed treatment in Vietnam and 76 (28%) by SAT within the United States.

### Losses along the LTBI Cascade of Care

Each point along the LTBI cascade of care saw losses in participation (Table 2, Table 3, Tables 4); 2,873 (54%) immigrant visa applicants approached for participation in PTOPS declined. Among eligible visa applicants who declined to participate, 881 (31%) noted they were too busy or stressed because of their impending move, 723 (25%) noted that either the study or the IGRA (or both) were not requirements for the medical examination, 641 (22%) noted their belief that they were not infected with *Mycobacterium tuberculosis*, 407 (14%) reported that family advised against enrollment, 178 (6%) reported concerns about blood draws, 37 (1%) noted concerns around delaying or otherwise affecting the visa process, and 27 (1%) noted their belief that prior Bacille Calmette-Guérin (BCG) vaccination would protect them against TB disease.

**Table 2:** Self-reported reasons for declining to participate in the Preventing Tuberculosis Overseas Pilot Study of US immigrant visa applicants, Vietnam, 2018–2019*

Reason†	No. (%) respondents
Total	2,873 (100)
Too busy or too much stress currently	881 (31)
Study or IGRA not required for medical examination	723 (25)
Did not believe infected	641 (22)
Family advised against enrollment	407 (14)
Worried about blood draw	178 (6)
Worried that participation could delay immigration process	37 (1)
Believed BCG vaccination would protect them from TB	27 (1)
Worried about enrolling in research	11 (<1)
Worried that IGRA results may affect immigration status	7 (<1)
Concerned about taking medication	6 (<1)
Worried about stigma	5 (<1)
Inconvenient to return to CRH VMD	5 (<1)
Did not understand study	1 (<1)
Undecided	1 (<1)

*BCG, Bacille Calmette-Guérin; CRH VMD, Cho Ray Hospital Visa Medical Department; IGRA, interferon-γ release assay; TB, tuberculosis. †A total of 2,940 reasons were given (*>*1 reason could be provided by respondents).

**Table 3:** Reasons IGRA not processed or 3HP not offered to participants in the Preventing Tuberculosis Overseas Pilot Study of US immigrant visa applicants, Vietnam, 2018–2019*

Reason	No. (%) participants
IGRA not processed for participants who consented to be tested	162 (100)
Previous TB or abnormality on chest radiograph	119 (73)
Hepatitis B	25 (15)
History of extrapulmonary TB	3 (2)
Previous treatment	1 (1)
Breastfeeding	1 (1)
Applying for visa type not included in study	2 (1)
Recent receipt of live virus vaccine	1 (1)
Unknown; may have withdrawn consent	10 (7)
3HP not offered to IGRA-positive participants	32 (100)
Hepatitis B	18 (56)
Hepatitis C	5 (16)
Previous TB or abnormality on chest radiograph	1 (3)
Liver disease	1 (3)
Planning to get pregnant in next 4 mo	1 (3)
Substance addiction	1 (3)
Previous LTBI treatment	3 (9)
Unknown	2 (6)

*IGRA, interferon-γ release assay; LTBI, latent tuberculosis infection; TB, tuberculosis; 3HP, 3-mo regimen of isoniazid and rifapentine.

**Table 4:** Reasons for declining to initiate 3HP and discontinuing treatment among participants in the Preventing Tuberculosis Overseas Pilot Study of US immigrant visa applicants, Vietnam, 2018–2019*

Reason	No. (%) participants
Declined to initiate 3HP	148 (100)
Not enough time; planned to depart for United States immediately after receiving visa	99 (67)
Preferred to take medicine in United States	23 (16)
Inconvenient to go to hospital for treatment: distance, time, or both	22 (15)
Concerned about adverse events from medicine	7 (5)
Did not feel sick	3 (2)
Treatment discontinued	36 (100)
Participant decided on own to stop because of grade 1 or 2 adverse events	18 (50)
Participant decided on own because too busy or moving to United States earlier	5 (14)
Identified as contact to a person with MDR or isoniazid-resistant TB or had extrapulmonary TB diagnosed after treatment initiation	5 (14)
Serious adverse event: grade 3 event, elevated liver function test, or both	5 (14)
Lost to follow-up in United States	3 (8)

*MDR, multidrug-resistant; TB, tuberculosis; 3HP, 3-mo regimen of isoniazid and rifapentine.

Of those who consented, 162 (7%) did not have their IGRA processed for study exclusions identified during the medical examination: 119 (73%) had an abnormal chest radiograph or another condition requiring further TB disease screening, 25 (15%) reported having hepatitis B, 3 (2%) had a prior history of extrapulmonary TB, 2 (<1%) were applying for a visa type that was not included in the study, 1 (<1%) previously completed LTBI treatment, 1 (<1%) was breastfeeding, and 1 (<1%) recently received a live-virus vaccine. For 10 (6%) persons, consent was withdrawn or the reason was not specified.

Of the 484 participants who were IGRA-positive, 32 (7%) were excluded on the basis of additional screening or laboratory results. Eighteen (56%) had hepatitis B, 5 (16%) had hepatitis C, 3 (9%) previously received LTBI treatment, 1 (3%) had liver disease, 1 (3%) had a substance addiction, 1 (3%) was planning to get pregnant in the next 4 months, 1 (3%) had an abnormal chest radiograph, and 2 (6%) participants did not specify the reason.

Of the 452 participants who were IGRA-positive and eligible for treatment, 148 (33%) declined treatment. Of those, 99 (67%) reported not having enough time for treatment because they were immigrating within 2 months, 23 (16%) preferred taking treatment in the United States, 22 (15%) thought weekly DOT at CRH VMD was inconvenient because of time or distance, 7 (5%) were concerned about adverse events, and 3 (2%) did not feel sick and therefore believed they did not need treatment.

Thirty-six (12%) persons who initiated treatment did not complete treatment. Eighteen (50%) did not want to continue because of a grade 1 or 2 adverse event, 5 (14%) suffered a serious adverse event or a grade 3 event resulting in treatment discontinuation, 5 (14%) were too busy to continue treatment or had to move earlier than anticipated, 5 (14%) were identified as contacts to persons with multidrug-resistant or isoniazid-resistant TB or were diagnosed with extrapulmonary TB after initiating LTBI treatment, and 3 (8%) were not available for follow-up after US arrival.

## Discussion

Our study suggests overseas (prearrival) LTBI testing and voluntary 3HP treatment during the required visa medical examination should be considered as a strategy to further US TB elimination efforts. Approximately 21% of all participants were IGRA-positive, and the proportion positive increased with age (24% of all adults); expanding IGRAs to adults could identify a high proportion of immigrants who have LTBI in Vietnam. We were able to achieve similar results for the proportion initiating LTBI treatment and a higher proportion for completion compared with current US postarrival efforts. In our study, 67% of eligible IGRA-positive participants initiated treatment, and 88% of those completed treatment, resulting in 59% of all eligible participants completing treatment. These results can be compared with a recent assessment of the recommended US postarrival evaluation for immigrants at risk for TB (2013–2016), in which 35.5% of immigrants and refugees at risk for TB did not complete a US postarrival evaluation for TB and LTBI; among those who did and were recommended for LTBI treatment, 69% initiated treatment and 40% completed treatment (*8*).

Currently, most low-incidence countries, similar to the United States, focus on postarrival strategies to address LTBI in immigrant populations (*16*,*17*). Although a few countries provide LTBI testing prearrival (*16*), our study evaluates offering voluntary LTBI testing and treatment to immigrants prearrival. A major challenge with postarrival screening for newly arriving immigrants and refugees is the lack of resources needed to follow up with recent arrivals to initiate and complete LTBI treatment (*18*). Proportions of postarrival follow-up have ranged from 60% to 75% over the years despite improvements, including the EDN system that alerts health departments to immigrants with a TB condition arriving in their jurisdictions (*14*,*19*). Further, because immune response tests, such as an IGRA, are not routinely required during the overseas examination for immigrants >15 years of age, those with LTBI are currently missed, and health departments therefore receive no alert from the EDN system of their arrival. However, expansion of just IGRA testing overseas, without also offering treatment overseas, would result in additional workload for already challenged health departments to follow-up and care for arriving adolescent and adult immigrants with LTBI. Moreover, immigrants themselves may experience challenges seeking care postarrival because of language barriers, transportation issues, and competing priorities with employment and educational commitments (*20*). These challenges and limitations underscore the need to maximize the use of the overseas process to improve LTBI testing and treatment among US-bound immigrants and refugees.

Our prearrival intervention demonstrated a high proportion of treatment completion, but for this approach to reach maximum effectiveness, 3 points along the cascade must be improved. First, participation and IGRA testing was low at 46%. Reported reasons for nonparticipation suggest that this low level was attributable to the perceived time commitment to participate in a study during a stressful time preparing for immigration to the United States (31%). Moreover, this project was conducted as a research study, coupled with a lengthy consent process, and whether this process itself deterred participation is unclear. Many visa applicants who declined to participate reported doing so because neither the study nor the IGRA was a requirement for immigration (25%). Thus, a decline for an IGRA was not necessarily caused by lack of interest in knowing one’s LTBI status. Currently, an IGRA is not a required element in the overseas medical examination for visa applicants >15 years of age and is not offered routinely to this age group. A prearrival IGRA would enhance the diagnostic workup for TB disease; routinely offering or requiring a prearrival IGRA for this group would have the added benefit of apprising immigrants of their TB infection status, giving them the opportunity to make an informed decision about LTBI treatment. Second, among those who learned they were IGRA-positive, treatment acceptance was 67%. Although this figure is similar to the proportion initiating treatment observed in the postarrival evaluation of immigrants and refugees with a B2 classification (mostly children) (*8*) in the United States and other studies evaluating 3HP (*21*), this proportion could be improved. Of participants who declined 3HP, 67% did so because they felt they did not have enough time to complete treatment before immigration. PTOPS participation required a minimum of 8 weekly DOT doses, meaning participants needed to remain in Vietnam for at least 2 months before immigrating to the United States. An additional 15% of participants declined treatment because of distance and time required to travel to CRH VMD for DOT. Third, although the proportion completing treatment was relatively high, 36 (12%) persons did discontinue treatment. Fifty percent of discontinuations were attributable to minor adverse events, and 28% were attributable to severe adverse events or other medical conditions resulting in treatment suspension. Fourteen percent of participants who discontinued treatment did so because they were too busy with their move to continue DOT. These data underscore the need for a strategy for LTBI testing and treatment that is person-centered, convenient, and not perceived by immigrants as interfering with their immigration plans.

Reducing the number of required DOT visits could theoretically increase treatment initiation and completion. This study included a minimum requirement of 8 weekly doses for DOT because, at the time, CDC recommended administration of 3HP by DOT. Now the World Health Organization and CDC have revised recommendations to support SAT for 3HP (*22*,*23*), enabling a reduction in the number of DOT visits. In addition, this course may be the least burdensome option for both staff and participants in terms of time and financial costs. However, although a SAT-only approach may increase treatment acceptance and completion for some persons, it may also result in more early treatment discontinuations because of concerns over minor side effects without the benefit of further support and education from healthcare workers during DOT visits. Because high rates of LTBI treatment completion are needed to be effective toward elimination (*24*) and missed appointments early in the course of treatment have been associated with completion failure (*25*), an approach worth considering is providing the first month of doses as DOT, using of digital adherence tools, or both, to allow participants to take their medicine and be supported without having to visit the clinic (*26*).

Recommendations for expanding overseas LTBI testing and treatment have been suggested previously (*11*); however, empirical evidence of how this approach would work has not been available. Until recently, diagnosis of LTBI relied upon the tuberculin skin test, which cross-reacts with BCG antigens. Thus, concerns existed about testing for infection because of the potential for false-positives in BCG-vaccinated populations. In addition, until recently, the standard LTBI treatment regimen was 9 months of isoniazid, a lengthy regimen prone to adverse events. The PTOPS approach relies on an IGRA, which is more specific than the tuberculin skin test, for diagnosis, reducing the potential for false-positive results (*27*). Moreover, PTOPS relies on voluntary acceptance of 3HP treatment. Although concerns exist that visa applicants may feel the need to comply with testing and treatment for immigration, our data suggest that visa applicants understood that testing (for those >15 years of age) and treatment were voluntary and that declining had no effect on immigration status (54% of applicants declined participation, and 33% of participants declined treatment). For the immigrant visa applicants, the PTOPS approach can be advantageous because it enables testing and treatment in a familiar environment and language and does not require participants to navigate the unfamiliar US healthcare system upon arrival.

The overseas medical examination is an opportunity to prevent importation of TB and contribute to elimination. This process has proven to be a high-yield intervention for identifying and treating TB disease in US-bound immigrants and refugees. Moreover, the successful implementation of the TB technical instructions (which included the addition of mycobacterial cultures and DOT for TB diagnosis and treatment) at the overseas panel physician sites (*10*) suggests that panel site personnel can acquire the necessary expertise to provide testing for TB infection and voluntary LTBI treatment (*28*). A cost-benefit analysis modeling implementation of LTBI testing and treatment at overseas refugees panel sites hypothesized that this approach could save millions of dollars compared with the current strategy of relying on post-arrival follow-up at health departments and could lead to a reduction of TB cases in the United States (*29*); however, a detailed evaluation of the actual costs and the benefits of this approach is needed. In addition, further studies should be conducted at other panel sites while also ensuring that visa applicants do not experience delays in migration.

Our study demonstrated that using the overseas medical examination to provide voluntary testing and treatment of LTBI in a high-burden country yields high initiation and completion of treatment and should be considered to address LTBI in US-bound immigrants and to advance TB elimination efforts. This strategy should be further evaluated as an addition to or replacement for post-arrival testing and treatment for LTBI and as a complement to other domestic strategies to address LTBI in immigrant populations.

More than 30 years have passed since the declaration of the US TB elimination goal and 20 years since the Institutes of Medicine published its report Ending Neglect: The Elimination of Tuberculosis in the United States. However, the basic question put forth in the report still remains: “[Will] the renewed opportunity that now presents itself to move toward the elimination of tuberculosis be seized or [will] tuberculosis be subject to another period of neglect until the next resurgence?” (*11*).
